# Comparison of Simulated Workplace Protection Factors Offered by N95 and P100 Filtering Facepiece and Elastomeric Half-Mask Respirators against Particles of 10 to 400 nm

**DOI:** 10.15436/2377-1372.15.015

**Published:** 2015-09-07

**Authors:** Xinjian He, Evanly Vo, M Horvatin, Y Liu, M Bergman, Z Zhuang

**Affiliations:** 1 National Institute for Occupational Safety and Health, National Personal Protective Technology Laboratory, Pittsburgh, PA; 2 URS, Inc. Pittsburgh, PA; 3 Industrial and Management Systems Engineering, College of Engineering and Mineral Resources, West Virginia University, Morgantown, WV; 4 Institute of Health Surveillance, Analysis and Protection, Hubei Center for Disease Control and Prevention, Wuhan, Hubei, China

**Keywords:** Nano particle, N95, P100, FFR, HER, SWPF, Respirator

## Abstract

This study compared the simulated workplace protection factors (SWPFs) between NIOSH-approved N95 respirators and P100 respirators, including two models of filtering facepiece respirator (FFR) and two models of elastomeric half-mask respirator (EHR), against sodium chloride particles (NaCl) in a range of 10 to 400 nm.

Twenty-five human test subjects performed modified OSHA fit test exercises in a controlled laboratory environment with the N95 respirators (two FFR models and two EHR models) and the P100 respirators (two FFRs and two EHRs). Two Scanning Mobility Particle Sizers (SMPS) were used to measure aerosol concentrations (in the 10–400 nm size range) inside (C_in_) and outside (C_out_) of the respirator, simultaneously. SWPF was calculated as the ratio of C_out_ to C_in_. The SWPF values obtained from the N95 respirators were then compared to those of the P100 respirators.

SWPFs were found to be significantly different (P<0.05) between N95 and P100 class respirators. The 10^th^, 25^th^, 50^th^, 75^th^ and 90^th^ percentiles of the SWPFs for the N95 respirators were much lower than those for the P100 models. The N95 respirators had 5^th^ percentiles of the SWPFs > 10. In contrast, the P100 class was able to generate 5^th^ percentiles SWPFs > 100. No significant difference was found in the SWPFs when tested against nano-size (10 to 100 nm) and large-size (100 to 400 nm) particles.

Overall, the findings suggest that the two FFRs and two EHRs with P100 class filters provide better performance than those with N95 filters against particles from 10 to 400 nm, supporting current OSHA and NIOSH recommendations.

## Introduction

Nanoparticles are defined as particles having at least one dimension between 1 and 100 nm^[1]^. Nanoparticles are produced by both natural (incidental nanoparticles) and industrial processes (engineered nanoparticles)^[2]^. Engineered nanoparticles are materials deliberately synthesized to have unique physical or chemical properties that allow them to be useful for specific applications. Recent studies have reported the presence of both incidental and engineered nanoparticles in a variety of workplaces^[3,4]^. Worker exposure to engineered nanoparticles in these workplaces is not well characterized although it has been suggested that generation and handling processes in industrial settings could generate aerosolized nanoparticles which might be inhaled, ingested or absorbed through skin^[5]^.

Respiratory protection for nanoparticles has been recommended by various organizations. However, there is no information available regarding what types of respirators should be required to use against nanoparticles. The lack of occupational exposure limits for many types of nanomaterials poses a significant challenge on selection of the most appropriate type of respirator^[6,7]^. The National Institute for Occupational Safety and Health (NIOSH), in its document “Approaches to Safe Nanotechnology–Managing the health and safety concerns associated with engineered materials”^[8]^, states that appropriate respirators can be selected based on the criteria described in the NIOSH Respirator Selection Logic^[9]^. The Occupational Safety and Health Administration (OSHA) recommends using the applicable General Industry standards for nanotechnology industries^[10]^. The U.S. Environmental Protection Agency (EPA) recommends NIOSH-approved respirators with an Assigned Protection Factor (APF) of at least 10 for nanoparticles such as siloxane-modified silica^[11]^. For single and multi-wall carbon nanotubes, however, the EPA recommendation specifies the use of NIOSH-approved tight-fitting air-purifying full-face piece respirators with N100 filters. The U.S. Department of Energy (DOE) recommends the use of half-mask respirators equipped with P100 cartridges/filters for airborne exposures of engineered nanomaterials^[12]^. Other organizations, including the International Standards Organization (ISO)^[13]^, have also recommended respiratory protection for workers exposed to nanoparticles.

After examining the use of respiratory protection in 82 nanoparticle manufacturing facilities internationally, it was reported that respirators were used at 22 of the facilities. P100 (FFR or cartridge) type respirators were stated as the most commonly reported type^[14]^. Dahm et al. found that elastomeric half-mask respirators (EHRs) were the most commonly used followed by FFRs after investigating 30 workplaces^[15]^. A number of studies have reported the filtration efficiency of NIOSH-approved respirators against nanoparticles^[16–18]^. It is acknowledged that the filtration efficiency of P100 rated filters is higher than that of N95 rated filters for nanoparticles. However, contribution from the faceseal leakage could be higher for P100 respirators (both FFR and EHR) due to higher breathing resistance (pressure drop) versus N95 respirators^[19]^, although there is some debate^[20]^. As a result, the performance for P100 respirators (i.e., any FFR or EHR with aP100 filter) may be similar to the N95 respirators^1^ if the respirator does not seal well. If this result is found it would motivate the need for design improvements for P100 FFRs to reduce faceseal leakage. Conversely, if P100 respirators are found to give higher protection then this would support recommendations to use a P100 class respirator. However, besides our recent submitted manuscript^[21]^, no data are available for comparing N95 versus P100 FFRs or EHRs using human subjects exposed to nanoparticle aerosols under simulated workplace activities.

The term simulated workplace protection factor (SWPF), defined as the ratio of ambient concentration of a given contaminant to that inside a respirator, is determined under laboratory conditions using test exercises designed to simulate work activities^[22]^. The SWPF takes into account particle penetration pathways such as filter media, faceseal leakage and leakage through the exhalation valve (if equipped) and any other components. Several studies investigated the SWPF for N95 FFRs^[23–26]^. The performance of twenty N95 FFRs and one elastomeric respirator was measured in a laboratory setting using a PortaCount Plus^[23,24]^. The 95^th^ percentile value for the total penetration for all the fit tested respirators combined was 4% (SWPF=25), which was higher than the OSHA APF for N95 class respirators (APF=10). Some researchers investigated the SWPF for particles < 100 nm for four different models of N95 FFRs using an Electrical Low Pressure Impactor (ELPI)^[27,28]^. The authors obtained the geometric mean protection factor (PF) of 21.5 for four commercially available N95 FFR models over the eight specific nanoparticle sizes. Nine samples of N95 FFRs for each model were tested and the PF values were <10 for at least one sample for each model, and the PF values were less for particles in the 40–200 nm range compared to the 200–1300 nm range, suggesting that the tested N95 FFRs failed to provide the adequate protection efficiency equivalent to the OSHA APFs against nanoparticles. On the other hand, although the filter media rating (N95 vs. P100) will not change the APF value for a given respirator type, it was recommended that, in the OSHA APF final rule discussion^[22]^, a respirator with a higher rated filter (e.g., upgrading from N95 to P100) should be chosen for workplaces with a large percentage of aerosols in the most penetrating particle size (MPPS) range^[7]^. For electret filter media (all the respirators tested in this study consist of this type of filter media) commonly used in today’s environments, the MPPS values are consistently < 200 nm (mostly in the range of 30 to 100 nm), which falls within the nanoparticle size region^[7]^.

It should be noted that this work was built upon our earlier effort^[21]^, which investigated performance of eight different respirators against nanoparticles under simulated workplace activities. Using the same data sets obtained from the earlier study, additional data analyses were performed with an overall objective to determine if P100 respirators (FFR and EHR) provide better performance than N95 respirators (FFR and EHR).

## Materials and Methods

### N95 and P100 Respirators

Two N95 FFRs (cup shape) from two manufacturers (company A and B) were chosen based on their design similarities. Two P100 FFRs (cup shape with exhalation valve) from the same two companies were selected as well. In addition, two N95 EHRs (one from company A and one from company B) and two P100 EHRs (one from A and one from B) were selected for testing. The total number of respirators selected was eight. All eight models are NIOSH-approved and commercially available. All EHRs are dual-filter designs.

### Experimental Design and Test Procedures

The experimental design and test procedures have been described in detail by Vo et al^[21]^. In brief, the experiments were carried out in a test chamber (2.5×1.5×2.5 m) in a controlled laboratory setting. Sodium chloride (NaCl) particles were generated using a six-jet atomizer (Model: 9036, TSI Inc.) and charge-equilibrated by passing them through a ^85^Kr electrical charge equilibrator (Model: 3054, TSI Inc.) prior to being released inside the test chamber. A NaCl particle concentration of ~2×10^5^ particles/cm^3^ was maintained during testing. Three mixing fans inside the chamber were used to circulate the NaCl aerosol. The challenge aerosol was log-normally distributed with a size range of 10–500 nm, a count median diameter of 60 nm, and a geometric standard deviation of 2.88.

The twenty five human test subjects were medically cleared for testing and gave their written consent to participate. The study was approved by the NIOSH Institutional Review Board. Before performing the SWPF test for a particular respirator model, the subject underwent a standard OSHA-accepted eight-exercise fit test using a Porta Count Pro+ (Model: 8038, TSI, Inc.). This fit test was performed to acquaint the subject with the donning and adjustment procedures and to ensure a good fit (i.e., passing fit factor ≥ 100) could be achieved. Passing fit factors were obtained for all test subject/respirator model combinations. After passing the PortaCount fit test, the test subject wearing the respirator entered the chamber and performed a set of six modified OSHA fit test exercises (3 min for each), which included 1) normal breathing, 2) deep breathing, 3) turning head side to side, 4) moving head up and down, 5) bending over, and 6) simulated reactor cleanout. The simulated reactor cleanout entailed the subject holding a scoop and performing a scooping motion from an empty bucket. Two Scanning Mobility Particle Sampling Systems (SMPS) (Model: 3080 EC with long DMA 3081, Model: 3772 CPC, TSI Inc.) were used to measure aerosol concentrations inside (C_in_) and outside (C_out_s) of the respirator, simultaneously. The SMPS measures particles in the 10–400 nm size range at a sampling flow rate of 0.6 L/min. SWPF was calculated as the ratio of C_out_ to C_in_ for each exercise. The overall SWPF was determined as follows:

$$SWPF=\frac{6}{\frac{1}{SWPF_{1}}+\frac{1}{SWPF_{2}}+\frac{1}{SWPF_{3}}+\frac{1}{SWPF_{4}}+\frac{1}{SWPF_{5}}+\frac{1}{SWPF_{6}}}$$

where SWPF_1_ through SWPF_6_ are the simulated workplace fit factors for exercises 1 through 6.

In this study, each subject made three visits to the laboratory. During each visit, all eight respirator models were donned and tested in predetermined random order. A total of 600 tests were performed: 25 (subjects) × 3 (visits) × 8 (models) = 600 tests.

### Data Analysis

SAS version 9.3 (SAS Institute Inc., Cary, NC, USA) was used for data analysis. Normality of the data was obtained by log_10_-transformation of the data points. Geometric mean SWPF was calculated for each respirator model, and for nanoparticles (10–100 nm) and large particles (100–400 nm) separately. Paired t-tests were performed to analyze the differences in SWPF between the N95 and P100 models of the same manufacturer and face piece type (either FFR or EHR). P-values < 0.05 were considered significant.

## Results

### N95 vs. P100, Filtering Facepiece Respirators (FFRs) SWPF against Nanoparticles (10–100 nm)

**Figure 1:**
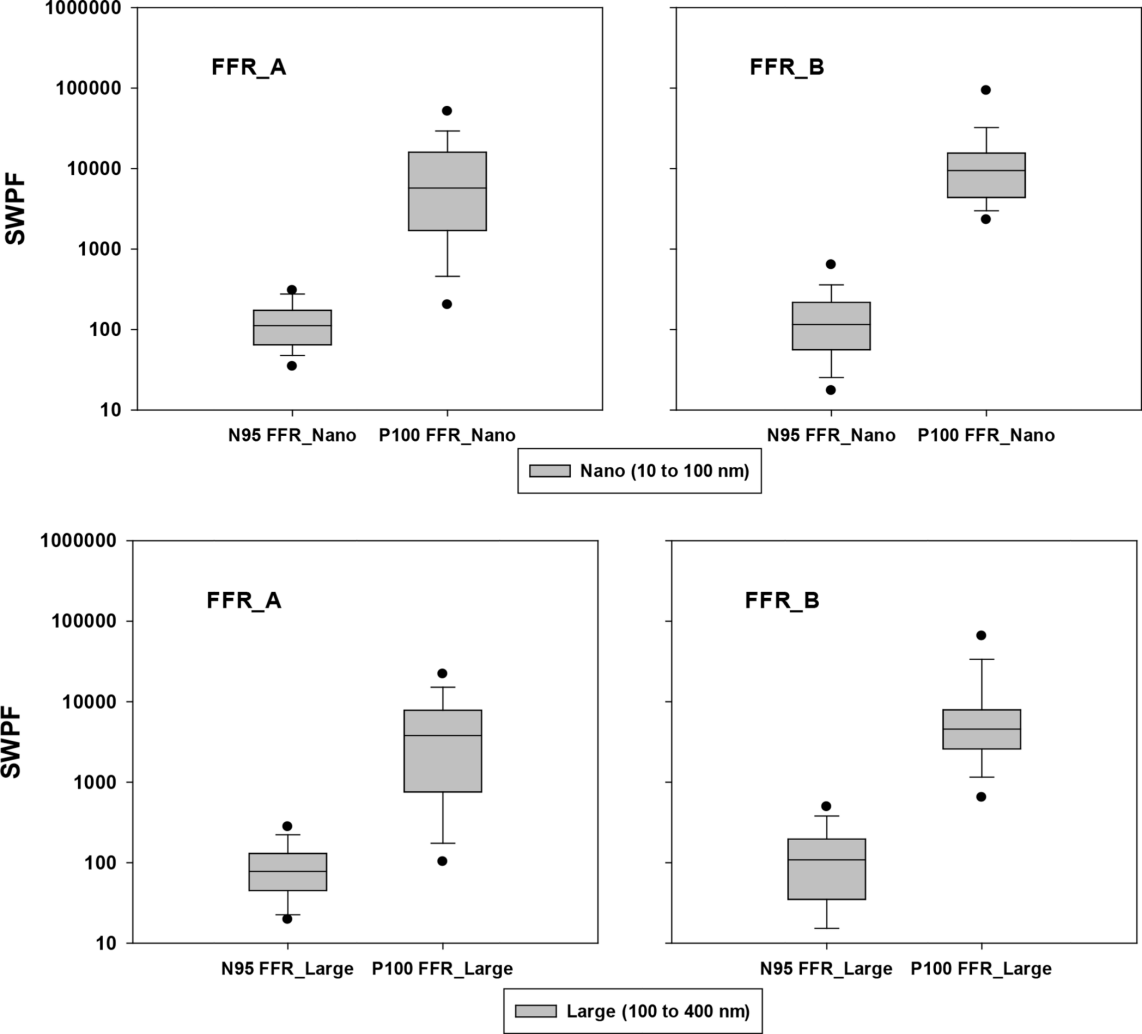
Simulated workplace protection factors (SWPF) offered by N95 and P100 filtering face piece respirators (FFRs)(from manufacturers A and B) against nano (10 – 100 nm) and large (100 – 400 nm) particles. Total observations are 73 for N95 FFR_A, 71 for P100 FFR_A, 73 for N95 FFR_B, and 73 for P100 FFR_B. The box plots show the following: dots (bottom and top) represent 5th and 95th percentiles; horizontal lines (from bottom) represent 10th, 25th, 50th, 75th and 90th percentiles.

[Figure 1] (top two panels) presents the SWPF values offered by the N95 and P100 FFR respirators (from manufacturers A and B) against nanoparticles (10 to 100 nm). For both FFR_A and FFR_B, the 10^th^, 25^th^, 50^th^, 75^th^ and 90^th^ percentiles of the SWPFs obtained from N95 FFRs were consistently lower than those produced by P100 FFRs. The 5^th^ percentiles of the SWPFs were > 10 for all tested N95 FFRs, which exceeds the OSHA APF of 10 for N95 FFR class respirators. All tested P100 FFRs provided remarkable protection against particles from 10 to 100 nm with the 5^th^ percentiles of the SWPFs >100, which was10-fold better compared to that of the N95 FFRs. The calculated geometric mean (GM) of the SWPFs was 110 for N95 FFR_A, 114 for N95 FFR_B, 4571 for P100 FFR_A, and 9420 for P100 FFR_B. Paired t-tests revealed that the difference in the SWPFs between the N95 FFRs and P100 FFRs was statistically significant (P < 0.05) for both FFR_A and FFR_B.

[Table 1] shows the percentage of subject-respirator combinations which had SWPFs below 10, 50, 100, and 200 against nanoparticles (10 to 100 nm). For example, no subject (0%) had a SWPF < 10 when wearing N95 FFR_A, 13.7% had SWPFs <50, 43.8% had SWPFs <100, and 82.2% had SWPFs < 200, whereas the corresponding percentages were 0% (<10), 20.5% (<50), 42.5% (< 100), and 72.6% (< 200) for the N95 FFR_B. In contrast, only 4.2% of subjects had SWPFs < 100, and all the subject-respirator combinations achieved SWPFs >200 for P100 FFR_B. Surprisingly, there was one subject (out of 71 subjects, 1.4%) who had a SWPF < 10 when wearing the P100 FFR_A. However, this abnormally low SWPF could be an outlier. It is noted that the total subject respirator combinations for each tested respirator model is less than 75 (25 subjects ×3 visits) due to a few missing data points or subjects dropping from the study.

### SWPF against Large Particles (100–400 nm)

The performance of the N95 and P100 FFRs was also evaluated against particles from 100 to 400 nm. The SWPFs offered by the tested FFRs are shown in [Figure 1] (bottom two). Similar to the findings obtained for nanoparticles (10 to 100 nm), the 10^th^, 25^th^, 50^th^, 75^th^ and 90^th^ percentiles of the SWPFs produced by N95 FFRs were consistently lower than those associated with P100 FFRs. As shown in [Table 2], it is clearly seen that lower percentages of subject-respirator combinations were identified for the P100 FFRs, suggesting that fewer subjects had SWPFs below certain values (e.g., 10, 50, 100, and 200) when wearing the P100 FFRs, except for the P100 FFR_A, which had a percentage of 1.4% (1 out of 71 subjects) that had a SWPF <10. Again, as mentioned above, this could be a result of an outlier. Statistical analyses (paired t-test) confirmed the significant difference (p<0.05) in SWPFs between the tested N95 FFRs and P100 FFRs.

### N95 vs. P100, Elastomeric Half-mask Respirators (EHRs) SWPF against Nanoparticles (10–100 nm)

**Figure 2:**
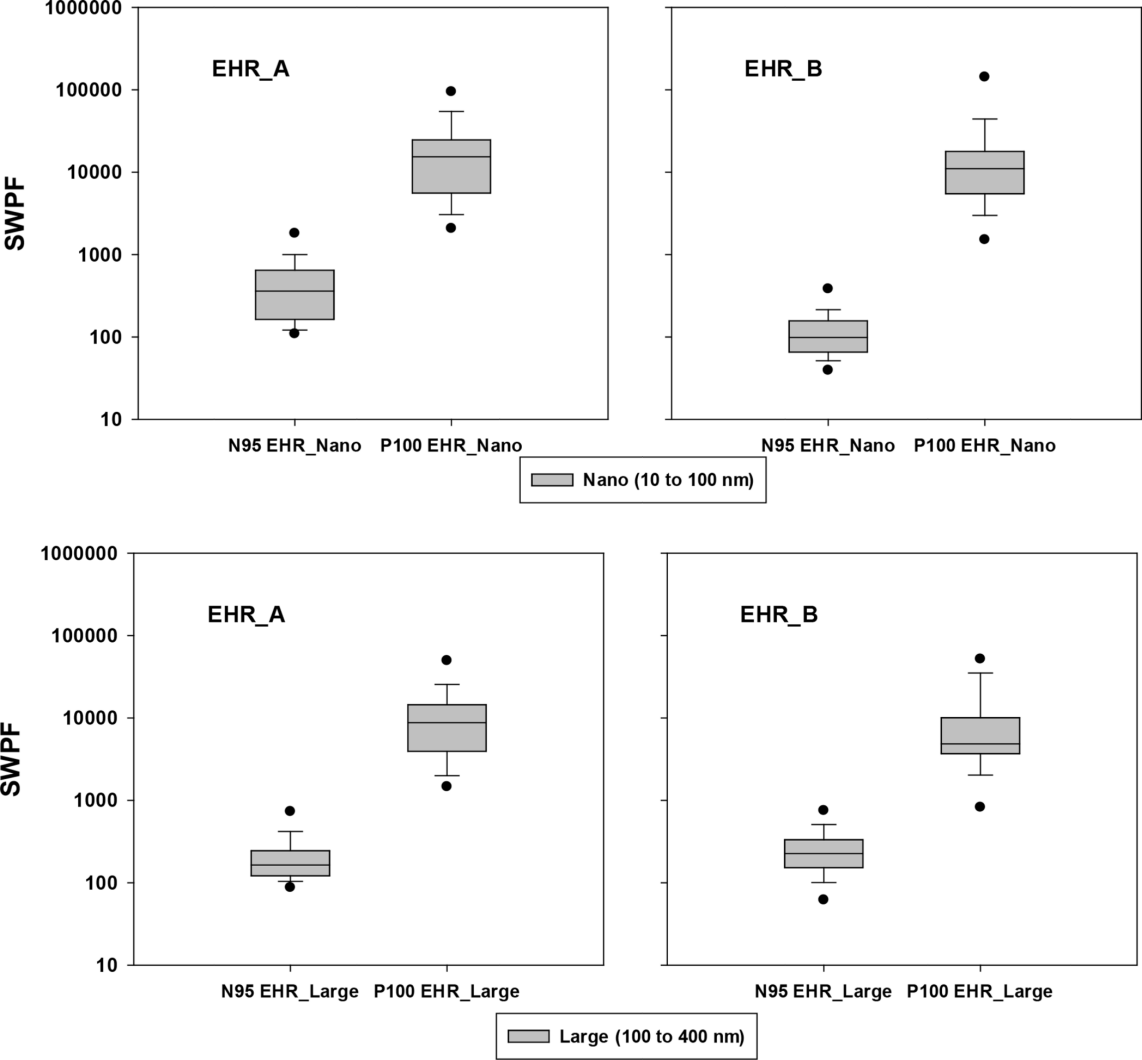
Simulated workplace protection factors (SWPF) offered by N95 and P100 elastomeric half-mask respirators (EHRs) (from manufacturers A and B) against nano (10 – 100 nm) and large (100 – 400 nm) particles. Total observations are 70 for N95 EHR_A, 70 for P100 EHR_A, 70 for N95 EHR_B, and 70 for P100 EHR_B. The box plots show the following: dots (bottom and top) represent 5^th^ and 95^th^ percentiles; horizontal lines (from bottom) represent 10^th^, 25^th^, 50^th^, 75^th^ and 90^th^ percentiles.

The SWPF values offered by the N95 and P100 EHRs against nanoparticles (10 to 100 nm) are presented in [Figure 2] (top two panels). The GM of the SWPFs calculated for the N95 EHRs were 358 (EHR_A) and 108 (EHR_B), whereas the GM values for P100 EHRs were 12605 (EHR_A) and 11046 (EHR_ B), respectively. Figure 2 also clearly shows that the 10^th^, 25^th^, 50^th^, 75^th^ and 90^th^ percentiles of the SWPFs for the N95 EHRs were much lower than those for the P100 EHRs (p<0.05, paired t-test). The 5^th^ percentiles of the SWPFs produced by the N95 EHRs were 111 (EHR_A) and 41 (EHR_B), which were higher than the OSHA APF of 10. On the other hand, the P100 EHRs were able to produce very high 5^th^ percentiles of the SWPFs (2344 for the EHR_A, and 1598 for the EHR_B).

Remarkably, all subject-respirator combinations of P100 EHRs achieved SWPFs >200 [See Table 1]. When the N95 EHRs were worn, 25.7% of subjects had SWPFs <200 for N95 EHR_A and 85.7% for N95 EHR_B. No subjects (0%) had SWPFs <10.

### SWPF against Large Particles (100–400 nm)

When used against particles from 100 to 400 nm, the P100 EHRs produced much higher SWPFs compared to N95 EHRs [See Figure 2, bottom two]. The calculated GM values were 7692 and 5977 for the P100 EHR_A and EHR_B, respectively, but only 188 and 218 for the N95 EHR_A and EHR_B, suggesting that the P100 EHRs offered significantly better protection (p<0.05, paired t-test) than the N95. This finding was also supported by the results listed in [Table 2]. All SWPFs produced by P100 were >200, whereas 67.1% and 44.3% of the SWPFs were below 200 for N95 respirators A and B.

## Discussion

Respiratory protection against exposures to engineered nanoparticles has been recommended by various organizations such as NIOSH, OSHA, EPA, ISO, etc. To date, no studies (besides our submitted work)^[21]^ have included both N95 and P100 respirators (FFR and EHR) to investigate SWPFs again-stnanoparticles. In our earlier effort^[21]^ several objectives were addressed: 1) measure simulated workplace protection factors (SWPFs) for both FFR and EHR types as a function of particle size; 2) determine if individual models within each type provide the expected level of protection; and 3) compare SWPF levels between class N95 and class P100 respirators and between FFR and EHR types. Only limited analysis of the third objective was performed in the previous work. In comparison, in this manuscript we reported the 5^th^, 10^th^, 25^th^, 50^th^, 75^th^, 90^th^ and 95^th^ percentiles offered by N95 vs P100 for FFR [Figure 1], and for EHR [Figure 2]. We also analyzed the percentage of data points below indicated SWPF (e.g., 10, 50, 100, and 200 nm) for particles of 10–100 nm [Table 1] and for particles of 100–400 nm [Table 2]. The corresponding discussion on those results were presented in the text body of the manuscript.

As the first of its kind, this study, along with our earlier effort^[21]^, revealed that the tested P100 respirators were generally able to provide at least 10 times better protection than the N95 when used against nano-size (10 to 100 nm) and large-size (100 to 400 nm) particles, which was true for both the FFR and EHR classes. This finding supports the current OSHA and NIOSH recommendations that a higher rated filter (e.g., changing from N95 to P100) should be chosen for workers to receive a better respiratory protection when exposed to a high percentage of nanoparticles in the workplace. Although the performance of the N95 respirators was less than the P100, the 5^th^ percentiles of the SWPFs were all greater than the OSHA APF of 10 for the tested N95 respirators; however, it is noted that the SWPFs were obtained under well-controlled laboratory conditions, thus the results may not represent the true protection levels in the field. In actual workplace environments involving higher work actives (higher work load), the protection levels would be expected to be lower^[27,29]^.

Though no study has compared N95 versus P100 respirators worn by human test subjects in a simulated workplace environment, several SWPF studies were conducted for various types of N95 respirators. Dulinget al. reported SWPFs of three types of respirators (N95 EHRs, N95 FFRs, and surgical masks) using a PortaCountPlus^[30]^. They found that 14% of the subject- respirator combinations had SWPFs < 10. However, when only subjects who passed a quantitative fit testing were included, all combinations showed SWPFs >10. Our results agree with the quoted study in that all tested N95 FFRs had SWPFs > 10. As mentioned in the methods section, all 25 human subjects passed a fit test screening before entering the exposure chamber for the SWPF test.

Fit testing is required under the OSHA Respiratory Protection Standard when tight-fitting respirators (including N95 and P100 FFRs and EHRs) are used^[31]^. However, not all workplaces are in compliance^[32]^. The importance of fit testing has been discussed elsewhere^[24,28]^. Coffey et al. demonstrated that when a fit test was applied to the test subjects, the 95^th^ percentile of total penetrations (equal to 1/SWPF) dropped from 33% to only 4%, and they concluded that fit-testing of N95 respirators is necessary to ensure the expected level of protection^[24]^. Another study conducted by Reponen et al. reported the same trend for N95 FFRs that 29% had PFs < 10 when all subjects were included, whereas 9% had PFs < 10 when only including the subjects who passed the fit testing^[18]^. While 9% of subject-respirator combinations had PFs < 10 in the latter quoted study, no subjects (0%) had SWPFs < 10 in this study. The possible factors causing the difference are different respirator models used (different fitting characteristics of the respirators), the size range of the challenge aerosols (0.04 to 1.3 µm in the quoted study), different sample flow rate (10 L/min in the referenced study), methods of measurement (ELPI used in their study), and subject variability (training received, motivation, age, facial characteristics etc.).

Another interesting finding of this study is that no statistical significance (P > 0.05) was identified in SWPF values against nanoparticles (10 to 100 nm) versus large particles (100 to 400 nm) after controlling the respirator model. Although several studies have demonstrated that total inward leakageis dependent on particle size^[29,33,–35]^ others found that the effect of particle size on TIL became less significant at lower flow rates (5–20 L/min)^[19,36]^. In addition, in this study the particles were-only separated into two sizes (nano-size from 10 to 100 nm, and large-size from 100 to 400 nm). Further data analysis would need to be performed to determine if other particle size ranges caused significant differences. However, this was not the goal of the current study.

There are a few limitations of this study. First, the SWPFs were measured under laboratory condition, which may overestimate the protection offered by the tested respirators. Our future study will determine if those respirators provide the expected level of protection for workers in real nanoparticle workplaces using portable size-selective instruments. The second limitation was the challenge aerosol (NaCl particles) used in the study, which may not be representative enough to reflect true particle characteristics (e.g., particle size, shape, density, charge, size distribution etc.). Therefore, field studies are necessary in the future to better understand the respiratory protection against various nanoparticles.

## Conclusions

The important finding was that the tested P100 FFRs and EHRs had significantly higher SWPFs compared to the N95 FFRs and EHRs, which is in agreement with our recent work^[21]^. Most of the N95 respirators achieved 5thpercentiles of the SWPFs > 10 when tested against nano-size (10 to 100 nm) and large-size (100 to 400 nm) particles, whereas the P100 class was able to generate 5^th^ percentile SWPFs > 100, which was tenfold greater than the N95 class. When the same respirator model was tested, it offered similar protection against nano-size and large-size particles. Overall, the findings suggest that the P100 respirators provide better performance than the N95 against particles from 10 to 400 nm, supporting current OSHA and NIOSH recommendations.

## Figures and Tables

**Table 1:** Percentage of data points below indicated SWPF_Nano for particles size range 10–100 nm

SWPF	N95 FFR_A	P100 FFR_A	N95 FFR_B	P100 FFR_B	N95 EHR_A	P100 EHR_A	N95 EHR_B	P100 EHR_B
	(n=73), %	(n=71), %	(n=73), %	(n=73), %	(n=70), %	(n=70), %	(n=70), %	(n=70), %
10	0	1.4	0	0	0	0	0	0
50	13.7	1.4	20.5	0	0	0	8.6	0
100	43.8	1.4	42.5	0	2.9	0	51.4	0
200	82.2	4.2	72.6	0	25.7	0	85.7	0

**Table 2:** Percentage of data points below indicated SWPF_Large for particles size range 100–400 nm

SWPF	N95 FFR_A	P100 FFR_A	N95 FFR_B	P100 FFR_B	N95 EHR_A	P100 EHR_A	N95 EHR_B	P100 EHR_B
	(n=73), %	(n=71), %	(n=73), %	(n=73), %	(n=70), %	(n=70), %	(n=70), %	(n=70), %
10	0	1.4	1.4	0	0	0	0	0
50	30.1	1.4	32.9	0	0	0	4.3	0
100	61.6	4.2	47.9	0	8.6	0	10	0
200	86.3	9.9	78.1	0	67.1	0	44.3	0

## Footnotes

1 **Notes:** In this manuscript, N95 respirators refer to filtering facepiece respirators (FFRs) or elastomeric half-mask respirators (EHRs) with N95 filters; similarly, P100 respirators refer to FFRs/EHRs with P100 filters.
